# A Comparative Analysis of Treatment Effects of PowerScope and AdvanSync2 in Class II Division 1 Malocclusion: A Retrospective Study

**DOI:** 10.7759/cureus.49105

**Published:** 2023-11-20

**Authors:** Avisha Middha, Yash D Shah, Seema Gupta, Farhan A Syed, Hemanth RV, Mohammed Nashiroddin

**Affiliations:** 1 Department of Orthodontics and Dentofacial Orthopaedics, Maharani Dental Clinic, Bikaner, IND; 2 Department of Oral Medicine and Radiology, Jawahar Medical Foundation's ACPM (Annasaheb Chudaman Patil Memorial) Dental College, Dhule, IND; 3 Department of Orthodontics, Jawahar Medical Foundation's ACPM (Annasaheb Chudaman Patil Memorial) Dental College, Dhule, IND; 4 Department of Orthodontics and Dentofacial Orthopaedics, Jal Mahal Dental Hospital, Jaipur, IND; 5 Department of Orthodontics and Dentofacial Orthopaedics, Partha Dental Skin Hair Clinic, Chennai, IND; 6 Department of Orthodontics, Al Badar Rural Dental College and Hospital, Kalaburagi, IND

**Keywords:** functional orthodontic appliances, dentoalveolar, skeletal, fixed orthodontic appliances, retrognathic mandible

## Abstract

Introduction: Fixed functional appliances are widely used for the treatment of retrognathic mandibles in skeletal class II malocclusion. The primary objective of the present investigation was to evaluate and compare the treatment effects induced by PowerScope (American Orthodontics, Sheboygan, WI) and AdvanSync2 (Ormco, Orange, CA). The hypothesis posited that there were no notable disparities in the therapeutic impacts of the two appliances.

Materials and methods: A retrospective study was undertaken involving 90 subjects with retrognathic mandibles. Group 1 was treated with AdvanSync2, group 2 received PowerScope treatment, and group 3 consisted of an untreated class II control sample. Lateral cephalograms were traced at pre-treatment (T0) and post-treatment (T1), to measure various skeletal, dental, and soft tissue parameters. The comparison between the groups was done using analysis of variance (ANOVA) and post-hoc analysis by Tukey's test.

Results: Significant changes were observed in all the parameters, comparing the effects of both appliances to the control group (p < 0.05). AdvanSync2 displayed statistically significant skeletal effects on the maxilla and mandible (p < 0.05). The statistically significant differences were also seen for dental effects such as reduction in overbite and overjet. On the other hand, PowerScope exhibited effects that were not considered statistically significant on the maxilla, instead primarily manifesting dento-alveolar changes that led to a considerable reduction in overbite. In comparison to the control group, both appliances notably produced soft tissue changes.

Conclusions: Our study rejected the null hypothesis. AdvanSync2 yielded superior skeletal outcomes with greater mandibular advancement, compared to PowerScope, which exhibited enhanced dento-alveolar alterations. AdvanSync2 took less treatment time, compared to PowerScope.

## Introduction

A retrognathic mandible is the most prevalent issue encountered in orthodontic clinical practice [1]. Fixed functional appliances (FFAs) are a viable alternative to removable functional appliances, especially when patients enter their final stages of development [1]. Various modifications of the Herbst and Forsus fatigue-resistant appliances are widely recognized as the most prevalent FFAs. An example of such an appliance is PowerScope, which was created by American Orthodontics (Sheboygan, WI) [2]. The drawback of this device is the necessity for complete alignment of the dental arches before placement, leading to non-utilization of residual growth in individuals [3-6]. To install the appliance, the process entails the positioning of stainless steel wires measuring 19 × 25 inches. This procedure necessitates six to nine months for proper treatment. The impact on the jaw growth caused by FFA exhibits variation contingent upon the age of the individuals. Individuals at the highest point of pubertal development display more evident skeletal consequences in comparison to those who are approaching the conclusion of the pubertal growth surge [7].

Recently, there has been the introduction of FFAs that are molar-to-molar [8]. AdvanSync2, developed by Ormco (Orange, CA), is one such device. Unlike other appliances, AdvanSync2 does not require prior alignment of arches or incorporation of rectangular stainless steel wires. As a result, it is possible to concurrently align arches while modifying growth, effectively utilizing an individual's residual growth [8]. A study conducted by Chitra et al. indicated that the utilization of AdvanSync2 during the post-pubertal stage led to significant changes in the profile of patients [9]. However, a limited number of studies have scrutinized the consequences of AdvanSync2 in the literature, predominantly consisting of instances of individual cases [8-11], and none have engaged in a comparative analysis of the repercussions of AdvanSync2 vis-à-vis a PowerScope appliance. Consequently, the principal objective of this investigation was to evaluate the dental, skeletal, and soft tissue effects of AdvanSync2 with those of PowerScope. The null hypothesis posited that there would be no detectable differences in the therapeutic effects between PowerScope and AdvanSync2.

## Materials and methods

Study design

This retrospective study was conducted on the records available in the Department of Orthodontics. Approval was obtained from the institutional ethics committee (SDCRI/IEC/2022/012A) before the start of the study. Written informed consent was obtained from all the patients to use their records for study purposes, without revealing their identity (anonymized data).

Sample size calculation

The sample size in this investigation was calculated using G*Power statistical software. The chosen parameters were a 95% confidence interval and beta error rate of 0.2. The standard deviation was set at 0.57, and the desired power was 0.90. Based on these parameters, the sample size was determined to be 25 per group [12]. This study included 30 patients in each group.

Eligibility criteria for the subjects

To select the study groups, a thorough evaluation of 225 patient records was performed, spanning from October 2020 to August 2022, and the study sample was chosen based on a set of specific eligibility criteria. The inclusion criteria included the subjects with skeletal class II (ANB angle ranging from 4° to 7°), a retrognathic mandible (SNB value < 77°), a positive visual treatment objective (VTO), subjects with full cusp or end-on molar relationships, minimal to moderate crowding of less than 4 mm, an overjet measurement ranging from 5 to 8 mm, an average growth pattern, and patients in stage 3 or 4 of cervical vertebrae maturation (CVM) [13]. Patients who presented with systemic problems, hormonal problems affecting growth, syndromic or craniofacial anomalies, previous history of trauma or temporomandibular joint disorders, missing teeth, cleft cases, and previous orthodontic treatment were excluded from the study. All records were examined to determine whether high-quality lateral cephalograms were available.

Group allocation

The study sample was divided as follows: group 1, AdvanSync2 consisting of 30 subjects (16 males, 14 females) with a mean age of 12.5 ± 1.4 years; group 2, PowerScope comprising 30 subjects (14 males, 16 females) with a mean age of 12.1 ± 1.2 years; and group 3, an untreated class II control sample of 30 subjects (15 males, 15 females), matched with the treatment groups in terms of skeletal age and observation period, with a mean age of 12.8 ± 0.8 years.

Intervention

**Figure 1:**
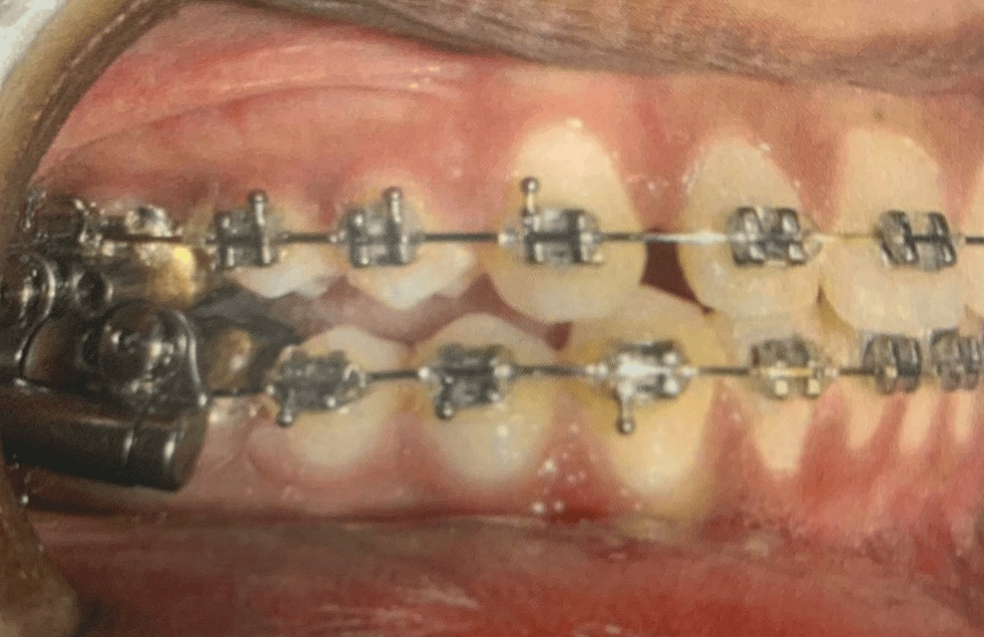
Intraoral view of AdvanSync2

**Figure 2:**
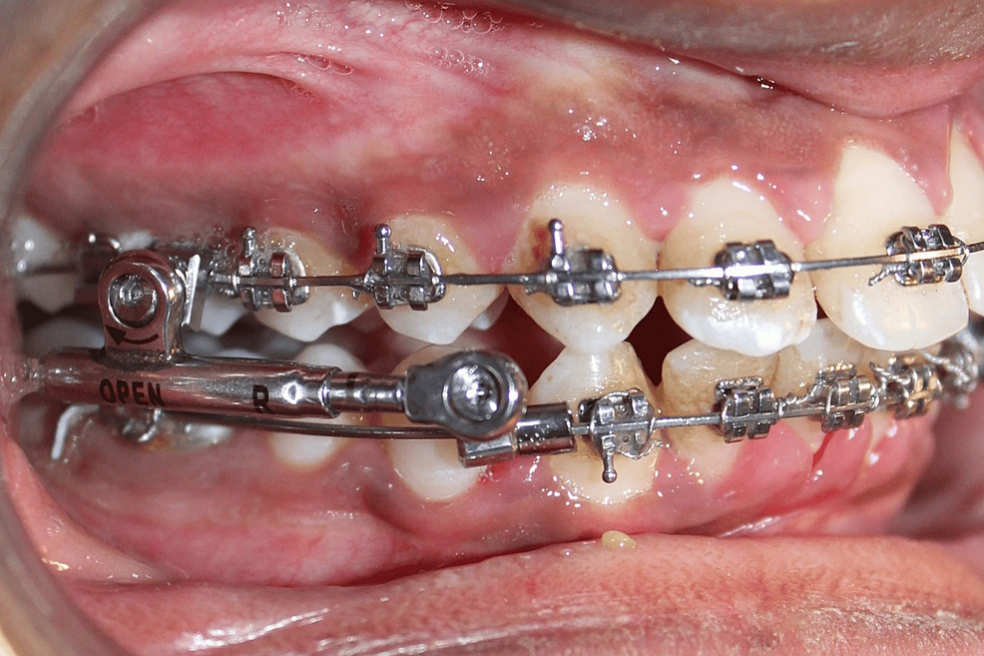
Intraoral view of PowerScope appliance

Two experienced orthodontists (SG, EB) with more than 10 years of experience administered treatment to all patients by employing a standardized treatment procedure. The treatment procedure for both groups entailed the use of fixed orthodontic treatment, with a 0.022 prescription. In the PowerScope cohort, preliminary leveling of arches was executed and systematically progressed to 19 × 25 in the stainless steel (SS) wire in all patients. This stage necessitated five to six months. Subsequently, the PowerScope apparatus was inserted as recommended and utilized in prior studies [2-4]. The technique employed in the AdvanSync2 group was similar to that reported by Dischinger [11]. The appliance is positioned during the initial bonding process. Before starting the treatment, lateral cephalograms were obtained (T0). Both the devices were gradually activated to attain desirable results, as shown in Figures 1, 2. Once the sagittal discrepancy was excessively rectified, any additional activation was discontinued, indicating termination of the established functional period.

Post-functional settling of occlusion and remaining treatment were completed in both groups. At this stage, lateral cephalograms were acquired for post-treatment records (T1) in both groups.

Outcome assessment

The lateral cephalograms at T0 and T1 were acquired in the natural head position using a KODAK 8000 C Digital Panoramic and Cephalometric system (Carestream Dental, Atlanta, GA), with an output of 90 KV and 8 MPa. The exposure parameters were consistent for all cephalograms. The individual responsible for tracing the cephalograms and the statistician were both unaware of the group assignment. The radiographs were meticulously traced using 25 measurements, as described by Jacobsen [14] and Riolo [15], which are presented in Table 1.

**Table 1:** Definitions of skeletal, dental, and soft tissue measurements These definitions are based on Jacobsen [14] and Riolo [15].

S. No.	Variable	Description
1.	SNA (°)	The angle between the SN plane and A point
2.	SNB (°)	The angle between the SN plane and B point
3.	ANB (°)	The angle between the point A to nasion (N) to point B
4.	SN-GoGn (°)	The mandibular plane angle between the SN plane and the gonion-gnathion plane (GoGn)
5.	U1-NA (°)	The angle between the long axis of the upper incisor and the NA plane
6.	L1-NB (°)	The angle between the long axis of the lower incisor and the NB plane
7.	IMPA (°)	The angle between the long axis of the lower incisor and the mandibular plane (MP)
8.	Z-angle (°)	The angle formed by Frankfort horizontal plane (FH) and profile line formed by touching the chin and most procumbent lip
9.	ANS-PNS ( mm)	The palatal plane formed between anterior nasal spine (ANS) and posterior nasal spine (PNS)
10,	Co-Gn (mm)	Mandibular length measured from condylion (Co) to gnathion (Gn)
11.	N-A-Pg (°)	Skeletal convexity measured from point nasion (N) to point A to pogonion (Pg)
12.	U1-NA (mm)	Linear distance between lower incisor (U1) to NA line
13.	L1-NB (mm)	Linear distance between lower incisor (L1) to NB line
14.	Overjet (mm)	The horizontal overlap of the upper incisors
15.	Overbite (mm)	The vertical overlap of the upper incisors
16.	UI-Palatal plane (PP) (mm)	The vertical distance between the upper incisor (U1) to the palatal plane (ANS-PNS)
17.	U6-Palatal plane (PP) (mm)	The vertical distance between the upper molar (U6) to the palatal plane (ANS-PNS)
18.	LI-GoGn (mm)	The vertical distance between the lower incisor (L1) to the gonion-gnathion plane (Go-Gn)
19.	L6-GoGn (vertical; mm)	The vertical distance between the lower molar (L6) to the gonion-gnathion plane (Go-Gn)
20.	U6-Ptv (mm)	The horizontal distance between the upper molar (U6) to the vertical pterygoid line (Ptv)
21.	L6-Ptv (mm)	The horizontal distance between the lower molar (L6) to the vertical pterygoid line (Ptv)
22.	Pg-Pg^’^ (mm)	Linear distance hard tissue pogonion (Pg) to soft tissue pogonion (Pg’)
23.	Nose prominence (Pr.) (mm)	The distance from a line perpendicular to the Frankfort horizontal and running tangent to the vermillion border of the upper lip, to the tip of the nose.
24.	E line-Upper lip (UL) (mm)	The linear distance from the upper lip to the E line, which is formed by touching the chin and the tip of the nose
25.	E line-Lower lip (LL) (mm)	The linear distance from the lower lip to the E line, which is formed by touching the chin and the tip of the nose

Method error

To determine the inaccuracies related to radiographic measurements, a random selection process was employed to select 30 cephalograms. Subsequently, these selected cephalograms underwent a meticulous procedure of retracing and remeasuring, conducted by the same investigator at a one-week interval. The method error (ME) was calculated using Dahlberg’s test. The ME was less than 1 for linear measurements (078 mm) and angular measurements (0.520). The intraclass correlation coefficient showed a high reliability of 94%.

Statistical analysis

The statistical analyses were performed using SPSS version 23 (IBM Corp., Armonk, NY). The Shapiro-Wilk test was used to assess the normality of the data. The data demonstrated a normal distribution for all variables except CVM stages. Consequently, parametric tests, such as analysis of variance and post-hoc analysis using Tukey's test, were employed to compare the mean differences among the three groups. To evaluate the mean differences in the CVM stages at T0 and T1 between the groups, a non-parametric Fisher's exact test was used. The level of significance was set at P ≤ 0.05.

## Results

Comparison of baseline characteristics

No significant differences were observed in the baseline characteristics between the groups. This was done to eliminate selection bias. At the beginning of the study (T0), non-significant differences were noticed in the ages of the participants (as measured by the CVM stages). However, as the study progressed from T0 to T1, significant differences were observed between the groups. Further examination of the CVM stage of patients in the PowerScope group at the pre-functional stage revealed that most patients were post-pubertal (CVM stage 5). There were significant differences in the total treatment time between the groups, as AdvanSync2 required less treatment time, compared to PowerScope, as shown in Table 2.

**Table 2:** Mean chronological ages, total time period, and CVMI stages at T0 (pre-treatment) and T1 (post-treatment) in the groups (ANOVA) CVMI: cervical vertebral maturation index; NS: non-significant; * p < 0.05: significant.

Parameters	AdvanSync2	PowerScope	Control	p-value
Chronological age at T0	12.5 ± 1.4 years	12.1 ± 1.2 years	12.8 ± 0.8 years	0.176^NS^
Chronological age at T1	14.23 ± 1.64 years	14.91 ± 1.24 years	14.82 ± 1.08 years	0.07^NS^
CVMI at T0	3.24	3.12	3.45	0.92^NS^
CVMI at T1	4.14	5.04	5.23	0.02^*^
Total time period (T0-T1)	1.23 ± 0.34 years	1.82 ± 0.92 years	1.88 ± 0.74 years	0.012^*^

All groups were comparable across all parameters, except for UI-NA (degrees) and LI-NB (degrees). Compared to the PowerScope and control groups, the AdvancSync2 group exhibited protruding anterior teeth during the pre-treatment stage, as depicted in Table 3.

**Table 3:** Comparability before treatment among the groups (ANOVA and Tukey test) SD: standard deviation; NS: non-significant; ** p < 0.01: moderately significant; *** p < 0.001: highly significant.

Parameters	Mean ± SD values			ANOVA test	Post hoc Tukey test		
	Group 1	Group 2	Group 3	p-value	1 vs 2	1 vs 3	2 vs 3
SNA (°)	80.36 ± 2.27	80.16 ± 1.91	79.60 ± 1.53	0.23^NS^	0.312	0.349	0.15
SNB (°)	74.92 ± 2.53	75.12 ± 1.92	74.80 ± 1.68	0.52^NS^	0.108	0.977	0.69
SN-GoGn (°)	27.28 ± 2.64	28.96 ± 2.55	28.96 ± 1.81	0.16^NS^	0.42	0.37	0.298
ANB (°)	5.44 ± 1.12	5.04 ± 1.69	5.20 ± 0.76	0.53^NS^	0.498	0.87	0.43
N-A-Pg (°)	4.76 ± 2.22	4.42 ± 1.384	4.64 ± 1.44	0.74^NS^	0.56	0.74	0.893
U1-NA (°)	32.12 ± 3.37	27.64 ± 6.83	30.44 ± 4.73	0.0002^***^	0	0.03	0.142
L1-NB (°)	29.76 ± 3.11	26.68 ± 4.27	26.80 ± 2.81	0.002^**^	0.006	0.009	0.991
IMPA (°)	103.28 ± 4.87	101.68 ± 8.54	101.64 ± 5.32	0.593^NS^	0.656	0.643	0.011
Overjet (mm)	6.36 ± 2.39	6.32 ± 2.11	6.04 ± 2.13	0.68^NS^	0.56	0.99	0.72
Overbite (mm)	2.93 ± 1.31	2.84 ± 1.45	2.34 ± 3.23	0.59^NS^	0.988	0.606	0.697
U1-PP (mm)	32.36 ± 2.09	32.45 ± 2.67	33.43 ± 3.43	0.82^NS^	0.26	0.34	0.431
LI-GoGn (mm)	25.42 ± 4.56	25.67 ± 2.31	25.67 ± 2.05	0.76^NS^	0.92	0.958	0.13
U6-PP (mm)	22.42 ± 1.087	22.42 ± 3.24	22.56 ± 2.09	0.62^NS^	0.91	0.15	0.18
L6-GoGn (mm)	28.13 ± 4.32	26.65 ± 2.45	28.23 ± 3.42	0.21^NS^	0.295	0.994	0.25
E line-UL (mm)	2.64 ± 1.58	2.22 ± 2.52	2.24 ± 1.56	0.15^NS^	0.32	0.33	0.936
E line-LL (mm)	-2.48 ± 1.56	-2.52 ± 1.80	-2.08 ± 1.50	0.57^NS^	0.995	0.66	0.606

Outcome measurement

Our study failed to accept the null hypothesis as statistically significant differences were observed between the groups, as shown in Table 4.

**Table 4:** Intergroup comparison of treatment changes (T1-T0) between the groups (ANOVA followed by Tukey test) T1: post-treatment; T0- pre-treatment; SD: standard deviation; NS: non-significant; * p < 0.05: significant; ** p < 0.01: moderately significant; *** p < 0.001: highly significant.

Parameters	Mean Difference (T1-T0) ± SD Value			ANOVA Test	Post hoc Tukey Test		
	Group 1	Group 2	Group 3	P value	1 vs 3	2 vs 3	1 vs 2
ANS-PNS (mm)	-0.49 ± -0.35	0.36 ± -0.21	0.72 ± -0.19	0.000^***^	0.0071^**^	0.123^NS^	0.188^NS^
SNA (°)	-0.56 ± -0.44	0.26 ± -0.40	0.56 ± 0.32	0.004^**^	0.000^***^	0.15^NS^	0.0015^**^
SNB (°)	2.36 ± -0.74	1.42 ± -0.51	0.41 ± -0.16	0.000^***^	0.000^***^	0.000^***^	0.008^**^
SN-GoGn (°)	0.12 ± -0.07	0.65 ± -0.17	0.16 ± -0.3	0.000^***^	0.756^NS^	0.000^***^	0.007^**^
Co-Gn (mm)	1.92 ± 0.07	1.96 ± -0.19	1.04 ± -0.48	0.000^***^	0.000^***^	0.000^***^	0.88^NS^
ANB (°)	-2.96 ± -0.53	-2.72 ± -1.06	-0.28 ± 0.07	0.000^***^	0.003^**^	0.005^**^	0.43^NS^
N-A-Pg (°)	-1.21 ± -0.57	-0.76 ± -0.49	-0.24 ± -0.29	0.000^***^	0.001^***^	0.002^**^	0.000^***^
U1-NA (°)	-3.04 ± -0.77	-3.76 ± -2.63	-0.48 ± -0.43	0.000^***^	0.002^**^	0.001^***^	0.256^NS^
UI-NA (mm)	-1.84 ± -0.79	-4.58 ± -0.89	-0.56 ± -0.32	0.000^***^	0.000^***^	0.000^***^	0.000^***^
L1-NB (°)	2.02 ± -0.3	2.92 ± -0.24	0.76 ± 0.15	0.000^***^	0.82^NS^	0.000^***^	0.000^***^
L1-NB (mm)	0.16 ± -0.51	1.72 ± -0.11	0.04 ± -0.27	0.000^***^	0.427^NS^	0.006^**^	0.000^***^
IMPA (°)	2.14 ± 0.97	2.36 ± 0.20	1.01 ± -0.32	0.000^***^	0.000^***^	0.000^***^	0.403^NS^
Overjet (mm)	-4.6 ± -1.24	-5.6 ± -0.88	-0.44 ± -0.42	0.000^***^	0.000^***^	0.000^***^	0.02^*^
Overbite (mm)	-0.02 ± -0.22	-1.50 ± -1.22	0.12 ± -0.89	0.000^***^	0.915^NS^	0.000^***^	0.002^**^
U1-PP (mm)	2.46 ± 1.05	-0.50 ± 0.24	0.33 ± -1.12	0.000^***^	0.009^**^	0.781^NS^	0.09^NS^
LI-GoGn (mm)	-0.26 ± 0.31	-2.13 ± 0.20	0.34 ± 1.73	0.000^***^	0.958^NS^	0.000^***^	0.000^***^
U6-PP (mm)	-1.64 ± 1.23	-0.69 ± -0.68	0.33 ± 1.03	0.000^***^	0.523^NS^	0.000^***^	0.001^***^
L6-GoGn (mm)	1.09 ± -0.8	1.87 ± -0.78	0.79 ± -0.53	0.000^***^	0.144^NS^	0.001^***^	0.004^**^
L6-Ptv (mm)	0.58 ± -0.11	1.32 ± -0.10	0.56 ± -0.23	0.000^***^	0.895^NS^	0.000^***^	0.006^**^
U6-Ptv (mm)	-1.75 ± -0.58	-1.08 ± -0.15	-0.28 ± -0.25	0.000^***^	0.000^***^	0.009^**^	0.009^**^
Pg-Pg’ (mm)	0.32 ± 0.48	0.52 ± 0.35	-0.24 ± -0.13	0.000^***^	0.708^NS^	0.016^*^	0.116^NS^
Nose Pr. (mm)	0.16 ± -0.68	0.93 ± -0.68	-0.24 ± -0.23	0.000^***^	0.873^NS^	0.002^**^	0.000^***^
E line-UL (mm)	-1.52 ± -0.31	-1.46 ± -0.29	-0.16 ± -0.27	0.000^***^	0.000^***^	0.000^***^	0.746^NS^
E line-LL (mm)	-1.98 ± -0.27	-1.26 ± -0.19	-0.28 ± -0.09	0.000^***^	0.000^***^	0.705^NS^	0.000^***^
Z angle (°)	-3.56 ± -0.41	-2.48 ± -0.82	0.60 ± -0.11	0.000^***^	0.000^***^	0.000^***^	0.023^*^

Skeletal effects

AdvanSync2 demonstrated notable effects on the maxilla, as evidenced by a decrease in SNA values and a constraint of 0.5 mm on the maxilla (p ≤ 0.05). In contrast, PowerScope exhibited effects on maxillary growth that did not reach statistical significance when compared with the control group. Despite both treatment cohorts exhibiting significant increases in mandible length (p ≤ 0.05) and notable advancement of the mandible (p ≤ 0.05) and enhancement of inter-jaw relationships, as seen by a reduction in ANB values and facial convexity (p ≤ 0.05), AdvancSync2 demonstrated superior effects compared to PowerScope. The opening of the mandibular plane angle was observed in group 2, whereas no notable discrepancy was observed in groups 1 and 3.

Dental effects

Groups 1 and 2 exhibited significant changes in the position of the teeth compared to group 3. The use of PowerScope resulted in a more pronounced backward inclination of the upper anteriors, forward inclination, and downward movement of the lower anteriors, as well as mesial movement and upward displacement of the lower molars. In contrast, the application of AdvanSync2 demonstrated greater distal and upward displacement of the upper molars, along with a more prominent downward displacement of the upper anterior teeth. Group 2 demonstrated a statistically significant reduction in the vertical and horizontal overlap of teeth compared with group 1.

Soft-tissue changes

A statistically significant increase in chin prominence, an increase in Z angle, and a reduction in the prominence of the upper lip were seen in groups 1 and 2 compared to group 3.

## Discussion

The objective of the present study was to assess the treatment effects induced by PowerScope and AdvanSync2 in comparison with untreated controls. The impetus driving this research was the dearth of literature pertaining to AdvanSync2 and the absence of any prior investigation comparing its impacts with those of PowerScope. Consequently, this study can be regarded as a significant enhancement to the current repository of knowledge. Existing evidence on FFA has revealed diverse treatment outcomes due to the absence of uniformity in baseline parameters among the different groups [7]. In the current investigation, noteworthy alterations in dentoskeletal and soft tissue characteristics were noted in both appliance groups compared to the untreated control group.

Skeletal changes

AdvanSync2 demonstrated a superior impact on skeletal outcomes compared with PowerScope. The effects of PowerScope on maxillary growth were not significant. This outcome aligns with the conclusions of Antony et al. [3]. AdvanSync2 exhibited a constraining effect on maxillary growth, as observed in a previous study [16]. This result is consistent with the findings of previous studies [8,10] but contradicts the observations made by Hemanth et al. [17]. These conflicting results may be attributed to variations in methodology, as the study was conducted on patients who had reached the postpubertal stage. Previous studies have reported that patients at the peak of puberty exhibited more notable changes in their skeletal structure than those who had reached post-puberty [10].

Both dental appliances achieved mandibular advancement; however, AdvanSync2 demonstrated a greater degree of advancement than PowerScope did. This finding was substantiated by previous research [10,18]. In contrast, some studies have reported contradictory results [8,19]. According to their findings, AdvanSync2 results in less mandibular advancement. It should be noted that these studies compared AdvanSync2 to other applications. A possible explanation for the enhanced skeletal effects generated by AdvanSync2 in our study may be its early implementation during the initial bonding phase of treatment, thereby making better use of the subjects' growth. In contrast, the use of PowerScope entailed the use of rectangular wires, which mandated several months of therapeutic intervention and yielded a reduction in the employment of the growth period in subjects [7,10]. As observed in our study, during the placement of the PowerScope, the majority of patients were post-pubertal at T1. Conversely, as a result of the application of AdvancSync2 during the preliminary stage of bonding, effective utilization of the growth period in the individuals was achieved.

Both cohorts demonstrated noteworthy enhancements in the inter-jaw correlation when juxtaposed with the control cohort. These changes were attributed to the advancement of the lower jaw and restricted growth of the upper jaw. This finding aligns with a previous study [20], which stated that FFA addresses skeletal class II by displacing the upper dental arch and pterygoid plate in backward and upward directions, while simultaneously protruding the lower jawbone and lower dental arch.

Dental changes

Both appliances exhibited significant alterations in dental structure compared to the control group. PowerScope demonstrated a greater degree of dental changes than AdvanSync2, which may be attributed to variations in the developmental stage of the participants. PowerScope exhibited a more pronounced reduction in overjet than AdvanSync2 owing to its superior capacity to retract the upper incisors [2,6]. The variance observed can be ascribed to the distally oriented forces applied to the maxilla by PowerScope, which exceed the forces exerted by AdvanSync2 [2]. These findings are in agreement with those of previous investigations that have assessed the dental ramifications of FFA [3,16,21]. AdvanSync2 exhibits a propensity for increased intrusion and distal movement of the upper molar teeth, which plausibly emanates from its placement [8]. Analogous findings have been documented in antecedent studies [8,19]. The vertical force plausibly engendered the intrusion of the upper molars and thus could be employed in patients with high-angle malocclusion.

A significant reduction in overbite was observed with the implementation of the PowerScope. This is in line with conclusions drawn from previous studies [2,5,6]. Conversely, the use of AdvanSync2 did not yield a significant correction for overbite. This discrepancy in outcomes may be related to the disparity in the manner in which the appliances were positioned. PowerScope utilizes an anterior attachment, whereas AdvanSync2 is situated on molars. Our findings contradict those of a previous study [18], which reported a substantial decrease in overbite with AdvanSync. It is advisable to employ FFA supported with mini plates to mitigate dental side effects on the lower anterior teeth [22].

Soft-tissue changes

Enhanced improvements in facial profile were observed following the administration of both appliances. The retraction of the upper lip, protrusion of the lower lip, elevation of the Z angle, and prominence of the chin collectively contributed to substantial improvement in the face of the patients. This improvement could be due to anterior positioning of the mandible, which was achieved through the use of FFA. These conclusions were corroborated by the findings of previous studies [23]. However, these findings contradict the conclusions reached in previous studies [12,17,24], where no notable alterations in soft tissue were detected after the administration of AdvanSync2. It is plausible that the dissimilarities in the patients' growth status may account for these incongruities [23].

Limitations of the study

The present study did not assess the alterations in treatment over an extended period nor did it examine any gender disparities. Consequently, future research endeavors must incorporate multicenter prospective studies involving diverse populations, to explore and evaluate the gender differences over a prolonged follow-up period.

## Conclusions

In the context of the treatment of skeletal class II patients, the preferred appliance is AdvanSync2 due to its prompt installation during the initial bonding phase. This appliance optimally harnesses the patients' growth potential and yields superior skeletal outcomes. Both devices engendered noteworthy treatment changes, thereby culminating in an enhancement of the facial profiles in the patients. AdvanSync2 exhibited superior skeletal modifications with restraining effects on the maxilla and a more pronounced mandibular advancement compared to PowerScope. On the contrary, the PowerScope device yielded more favorable outcomes in terms of dental effects.
